# Supplementary material for: Regulation of Fibroblast Activation Protein by Transforming Growth Factor Beta-1 in Glioblastoma Microenvironment
Source: Int J Mol Sci. 2021 Jan 21;22(3):1046. doi: 10.3390/ijms22031046 (PMC7864518; doi:10.3390/ijms22031046)
Supplement: Supplementary file 1 [file ijms-22-01046-s001.pdf]

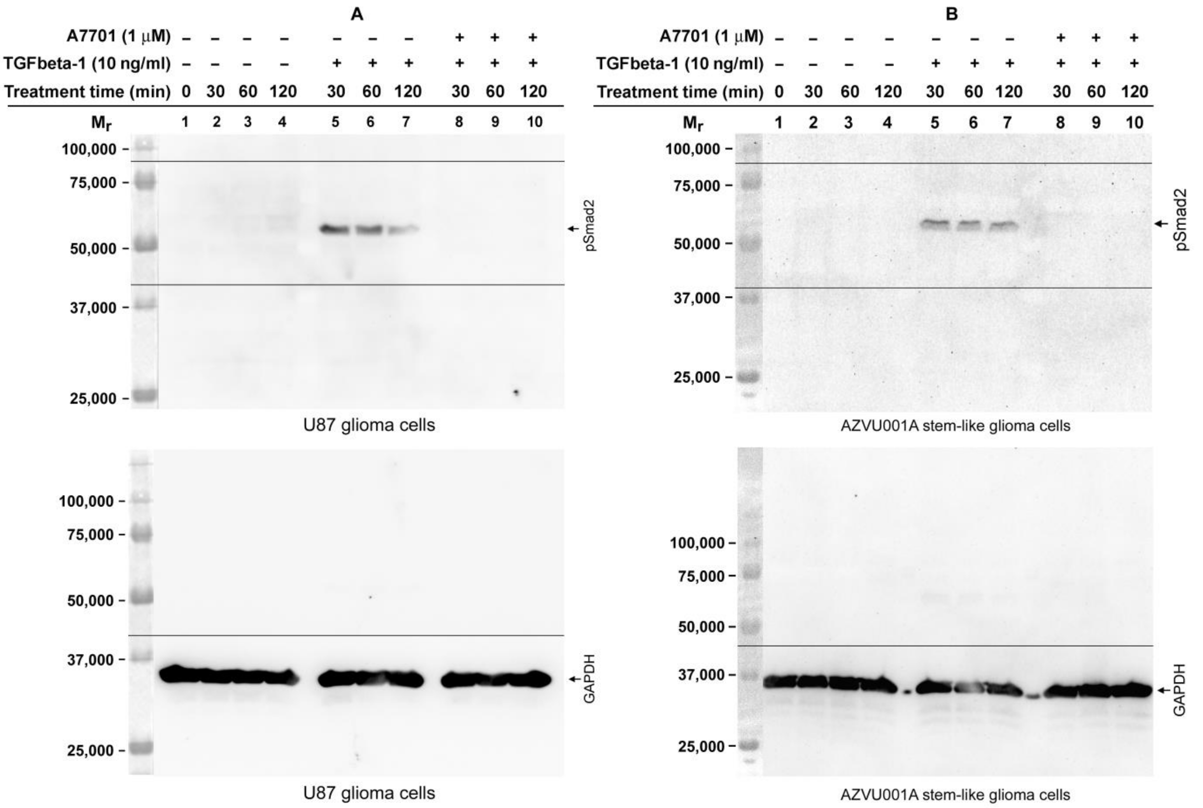

Figure 1S. Western blot analysis of TGFbeta-1-induced phosphorylation of Smad2 protein in glioma cells**.** Source images (A) and (B) showing the full length immunoblotting membranes after the detection of pSmad2 (upper halves) and GAPDH (lower halves) proteins in the TGFbeta-1 treated FAP-upregulating U87 glioma cells and FAP-non-upregulating AZVU001A glioma stem-like cells. These images were cropped to obtain the respective Figures 8A and 8B. The cropping lines are shown.
